# Obesity pharmacotherapy in older adults: a narrative review of evidence

**DOI:** 10.1038/s41366-024-01529-z

**Published:** 2024-05-06

**Authors:** Alex E. Henney, John P. H. Wilding, Uazman Alam, Daniel J. Cuthbertson

**Affiliations:** 1 Department of Cardiovascular & Metabolic Medicine, University of Liverpool, Liverpool, UK; 2 Metabolism & Nutrition Research Group, Liverpool University Hospitals NHS Foundation Trust, Liverpool, Merseyside, UK; 3 Liverpool Centre for Cardiovascular Sciences, University of Liverpool and Liverpool University Hospitals NHS Foundation Trust, Liverpool, Merseyside, UK

**Keywords:** Obesity, Weight management, Epidemiology

## Abstract

The prevalence of obesity in older adults (people aged >60 years) is increasing in line with the demographic shift in global populations. Despite knowledge of obesity-related complications in younger adults (increased risk of type 2 diabetes, liver and cardiovascular disease and malignancy), these considerations may be outweighed, in older adults, by concerns regarding weight-loss induced reduction in skeletal muscle and bone mass, and the awareness of the ‘obesity paradox’. Obesity in the elderly contributes to various obesity-related complications from cardiometabolic disease and cancer, to functional decline, worsening cognition, and quality of life, that will have already suffered an age-related decline. Lifestyle interventions remain the cornerstone of obesity management in older adults, with emphasis on resistance training for muscle strength and bone mineral density preservation. However, in older adults with obesity refractory to lifestyle strategies, pharmacotherapy, using anti-obesity medicines (AOMs), can be a useful adjunct. Recent evidence suggests that intentional weight loss in older adults with overweight and obesity is effective and safe, hence a diminishing reluctance to use AOMs in this more vulnerable population. Despite nine AOMs being currently approved for the treatment of obesity, limited clinical trial evidence in older adults predominantly focuses on incretin therapy with glucagon-like peptide-1 receptor agonists (liraglutide, semaglutide, and tirzepatide). AOMs enhance weight loss and reduce cardiometabolic events, while maintaining muscle mass. Future randomised controlled trials should specifically evaluate the effectiveness of novel AOMs for long-term weight management in older adults with obesity, carefully considering the impact on body composition and functional ability, as well as health economics.

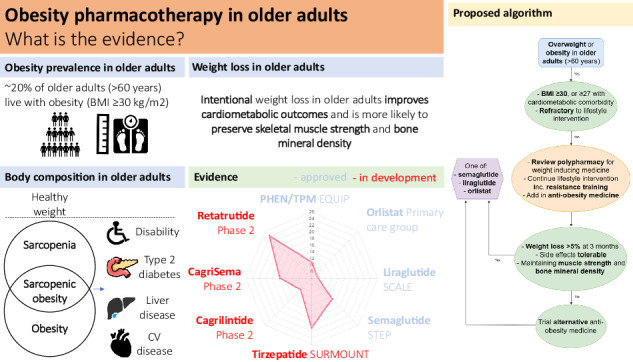

## Introduction

Although ageing populations highlight the success of modern health care, many people living into older age, commonly considered >60 years [1], have impaired quality of life (QoL) attributable to physical disability and/or a variety of chronic diseases [2]. Obesity, poor diet quality, and sedentariness have become more prevalent, whilst smoking has declined [3, 4]. The biggest increase in prevalent obesity has been in older adults, with ~20% of this age group now living with a body mass index (BMI) ⩾30 kg/m^2^ [5].

The ‘obesity paradox’ suggests that increasing adiposity in older adults is paradoxically associated with a lower mortality risk, and thus obesity has been considered less concerning in this population [6]. It is believed this potential protective effect of living with obesity in older adults is restricted to those living only with overweight/lesser degrees of obesity [7], hence the rationale for pharmacological intervention to treat excess weight in older adults is a relevant therapeutic consideration as it would be in younger people.

We are currently amid an evolving revolution in obesity pharmacotherapy, with increasingly efficacious anti-obesity medicines (AOMs), incorporating uni-, bi- and trimolecular incretin receptor agonists [8, 9]. Our improved understanding of the complex biological regulation of appetite, metabolism and body weight, involving co-ordinated responses between peripheral tissues and central appetite-regulating centres, has resulted in the availability of nine Food and Drug Administration (FDA) (liraglutide, semaglutide, tirzepatide, orlistat, phentermine, phentermine/topiramate, bupropion-naltrexone, setmelanotide, and metreleptin), and seven European Medicine Agency (EMA) (excluding phentermine and phentermine/topiramate), approved AOMs. However, scarce evidence exists for these AOMs in older adults [10], hence the current narrative will review the evidence for obesity pharmacotherapy in this sub-population.

## Age-related changes in body composition and their sequelae

**Fig. 1:**
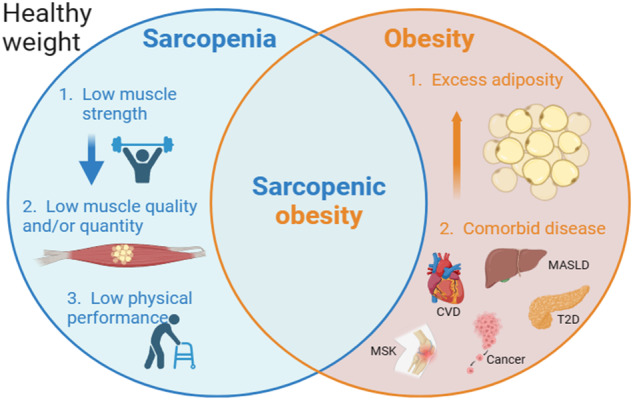
Venn diagram exploring the four body composite phenotypes in older adults (including healthy weight). The triad of sarcopenia, as defined by The European Working Group on Sarcopenia in Older People (EWGSOP), is illustrated [35]. Co-morbid complications related to obesity include, but are not limited to, cardiovascular disease (CVD), metabolic dysfunction associated steatotic liver disease (MASLD), type 2 diabetes (T2D), hormone-dependant cancers, and musculoskeletal (MSK) issues such as osteoarthritis.

Progressive change in body composition occurs with ageing, characterised by four phenotypes in older adults, that can increase the likelihood of mortality through comorbid disease [11]: sarcopenia, healthy weight, obesity and sarcopenic obesity [12] (Fig. 1).

*Obesity* is a chronic relapsing disease process, attributable to the complex interaction between genes and environmental risk factors, characterised by excess adiposity, that adversely impairs health [13]. Obesity is equally defined in older and younger adults using a BMI ⩾30 kg/m^2^, with ethnic specific BMI thresholds [14], although surrogate markers of fat mass and distribution, such as waist circumference (WC) and height-to-weight ratio, may more accurately define body composition in older adults [15]. People living longer with obesity may have associated comorbidity [16], involving musculoskeletal, cardiometabolic and mental health complications, with higher rates of malignancy [17], although it remains unclear the extent to which cumulative exposure to obesity impacts upon their respective clinical outcomes [18–21]. It has been postulated that early-onset obesity carries favourable adipose distribution, conferring a metabolically healthier phenotype than if obesity develops in older adults [20].

### Quality of life

Physical disability typically develops once the BMI exceeds 30 kg/m^2^ [22], with negative impact on QoL [11]. Chronic pain is the main driver of obesity-related disability, explained by increased mechanical load, low-grade inflammation and consequences of cardiometabolic disease (peripheral neuropathy and claudication) [23].

### Cardiometabolic disease

The prevalence of T2D in older adults correlates with BMI and WC [24, 25]. Five distinct clusters of diabetes have been proposed, of which mild age-related diabetes is the most common, making up 40% of diabetes diagnoses, and is directly attributable to ageing-related ectopic fat deposition and insulin resistance [26]. The impact of cumulative exposure to, and age-of-onset of, obesity on diabetes incidence is unclear [18–21]. Similarly, the association between obesity with cardiovascular disease (CVD), and cognitive decline, in older adults is not definitive. Recent prospectively designed studies have found higher WC increases CVD risk in older adults [27, 28], whilst meta-analyses demonstrate increased risk of incident dementia following obesity in mid-life, but a reduced risk when obesity is developed in older adulthood [29, 30].

### Cancer

In post-menopausal women, obesity is associated with breast cancer, malignant melanoma, and endometrial cancer [31, 32], whilst both older males and females are at increased risk of hepatobiliary (gallbladder and pancreatic), and genitourinary (renal, bladder, uterine, cervical and prostate) cancers [33].

### Mortality

The obesity paradox suggests older adults living with obesity are relatively protected against mortality compared to their younger counterparts [6]. However, evidence suggests that there is a divergence point, related to obesity severity, at which mortality risk becomes increasingly modulated. Thus the evidence would suggest that although overweight may reduce mortality risk, obesity clearly and incrementally increases mortality risk (vs. healthy weight) [7]. Crucially, the obesity paradox is likely heavily confounded by two factors: (1) whether weight loss is intentional, and (2) the use of the BMI metric, which poorly reflects the contribution of body composition towards body weight in older adults [15, 34]. Moreover, another meta-analysis concludes that older populations, being overweight was not found to be associated with an increased risk of mortality [16].

#### Sarcopenia

The European Working Group on Sarcopenia in Older People (EWGSOP) describe a triad for sarcopenia diagnosis: (1) low muscle strength, (2) low muscle quantity/quality, and (3) low physical performance. EWGSOP advocate a primary care assessment combining a questionnaire (SARC-F; a 5-item questionnaire relating self-reported clinical features of sarcopenia) with muscle strength testing, such as grip strength, and suggest more detailed assessment using image-derived quantification of muscle quantity/quality, and physical performance testing, are reserved for disease prognostication and research purposes [35]. Sarcopenia aetiology is multi-factorial, driven by progressive anabolic resistance to nutrition and physical activity [36, 37], and age-related structural (vertebral compression and unfavourable fat deposition [38]), metabolic (reduced basal metabolic rate [39]) and hormonal (lower circulating anabolic hormones (testosterone, growth hormone and insulin-like growth factor-1)) changes; more prominent in adults of lower socioeconomic status [40].

As with obesity, reduced muscle strength in sarcopenia is associated with disability and poor QoL [25], as well as risk of adverse events and subsequent hospital care, increasing the financial burden to the health service [41]. Moreover, a recent meta-analysis demonstrates significantly higher mortality risk in patients living with sarcopenia [42].

#### Sarcopenic obesity

Finally, the confluence of ageing and obesity epidemics has resulted in the concomitant presence of accelerated sarcopenia and obesity, termed sarcopenic obesity, which seems to be associated with a risk synergistic with those of the individual entities [12].

## Impact of weight loss on older adults

Weight loss in younger adults brings about multiple benefits. Weight loss of 5–10% total body weight can improve physical function and such cardiometabolic risk factors as blood pressure and lipid profile, whilst weight loss exceeding 10% can additionally improve cancer risk, fertility, and health-care costs, while further improving cardiometabolic outcomes [43, 44]. The same body weight, dose-response relationship cannot be assumed to hold true for older adults, and the risk of sarcopenia and reduced bone density with significant weight loss needs to be considered. However, many epidemiologic studies fail to account for the intentionality of weight loss. Unintentional weight loss, more likely driven by comorbid disease, excessively impacts metabolically active tissue over fat mass [45]. As a result, data are heterogenous, with beneficial effects of weight loss on disability and cardiometabolic health, but also important risks that may be forgotten about by clinicians.

### Morbidity

The comorbid impact of weight loss on older adults can be collectively considered by its musculoskeletal and metabolic impact with physiological effects on different tissues such as skeletal muscle and bone, and on cardiovascular and metabolic function.

#### Skeletal muscle

Generally, each kilogram of body weight lost constitutes ~75% fat mass and ~25% muscle mass [46], although the relationship is non-linear and governed by the magnitude of weight loss [47]. When older adults lose weight under supervision, their physical function and QoL outcomes improve [48, 49]. Concomitantly, muscle mass is more likely preserved during intentional weight loss, but is reduced during unintentional weight loss [50]. Interestingly, loss of muscle mass in itself appears not to be associated with functional decline and disability [51] but rather maintenance of muscle strength is key to preventing functional decline [52], with randomised controlled trials (RCTs) demonstrating the benefit of resistance training in older adults [53]. Muscle strength and muscle fat infiltration are inversely correlated [54], and it may be that targeted weight loss and resistance training improves functionality through favourable structural muscle remodelling [46].

#### Bone

Two reviews have highlighted the impact of weight loss on reducing bone mineral density and risk of fractures, which may reduce mobility [50, 55]. Every one kilogram reduction in body weight is associated with a decrease in bone mineral density of 0.1% at the femoral neck [50]. However, a RCT of 187 older adults with obesity and cardiometabolic disease found that resistance training can counteract the reduced bone mineral density following weight loss [56].

#### Cardiometabolic disease

Observational studies demonstrating associations between weight loss in older adults and adverse cardiometabolic outcomes, such as CVD and dementia, fail to discern the intentionality of weight loss [57, 58]. When weight loss is intentional, older adults significantly improve cardiometabolic risk markers [59, 60]. One RCT demonstrated improved cardiometabolic risk markers in older adults losing 8% body weight from baseline with energy-restriction and exercise (vs. placebo) [61]. Another trial randomised older adults to healthy eating, hypocaloric diet, or very-low energy diet (VLED) groups, found that all diets improved cardiometabolic risk markers and reduced the number of people requiring T2D and CVD prevention medication, especially with VLEDs [62]. Intentional weight loss of 3–4 kg over 1 year in older adults improves glucose tolerance, incident T2D and CVD [55].

### Mortality

A meta-analysis of observational studies in older adults raised concerns regarding weight loss and mortality outcomes. Weight loss was associated with a 59% increase in mortality risk. However, weight gain also increased all-cause mortality by 10%, and the analysis again failed to account for intentionality of weight loss [63]. Conversely, a cohort study using standard dietetic advice to produce intentional weight loss in older adults with T2D demonstrated that every 1 kg of weight loss was an associated 3–4 months survival prolongation [64], and a meta-analysis of RCTs assessing the impact of intentional weight loss on mortality found a 15%, although the mean age of participants was 52 years [65].

Overall, intentional weight loss in older adults appears safe, and may provide benefit to physical function, disability, QoL and cardiometabolic outcomes, when done under supervision of clinicians.

## Current treatment strategies for obesity in older adults

**Fig. 2:**
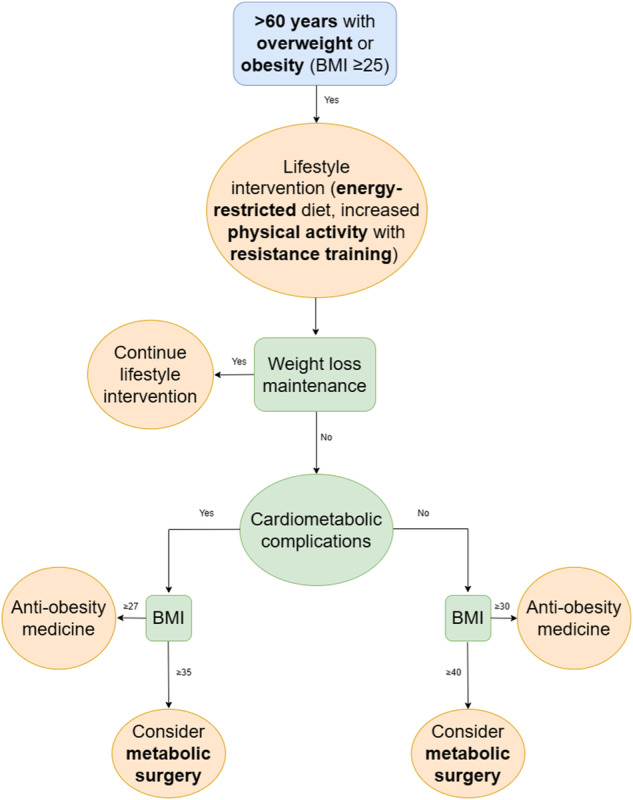
General algorithm for the management of obesity including lifestyle intervention, pharmacological therapy, and metabolic surgery. BMI body mass index.

Weight loss interventions may consist of lifestyle, pharmacological and surgical approaches, implemented in a stepwise approach (Fig. 2).

### Lifestyle intervention

Energy-restricted diet, increased physical activity and eating behaviour modification, delivered through behavioural weight management programmes (BWMPs), are the mainstay of obesity management in older adults. Several RCTs have investigated the weight loss efficacy of lifestyle interventions in this population. Dietary intervention produces greater weight loss, whereas physical activity produces better physical function, although the combination of interventions provides superior improvement in physical function than either intervention alone [66]. Therefore, emphasis should be placed on combining energy-restriction with a high protein diet and physical activity, to provide the maximal anabolic stimulus, ensuring weight loss is not associated with loss of muscle mass, or, more importantly, muscle strength [67, 68]. Sufficient dietary protein intake during weight loss in older adults may be achieved using whey protein supplementation [69]. Importantly, older adults adhere better to, and maintain greater weight loss from, lifestyle interventions [70–72], and in the real-world setting, the National Institute for Health and Care Excellence (NICE) report that older participants lose more weight than younger adults during BWMPs [73]. This aligns with knowledge that older people are well motivated to lose weight and engage with weight management services [74].

### Obesity pharmacotherapy

AOMs can be initiated when weight loss is refractory to lifestyle interventions. Weight loss exceeding 10% is needed for improvement in cardiometabolic outcomes [43], which is the main motivation older adults cite for losing weight [73]. Novel AOMs, largely based around incretin therapy, may exceed weight loss of 20% [10, 75], however, most trials evaluating their efficacy and safety were performed with younger adults, and those that include older adults often poorly define the number of participants in each age group. When prescribing AOMs in older adults, consideration of drug-drug interactions should be sought in light of polypharmacy [76], as well as side effects, either directly through the pharmacological properties of the AOM, or indirectly through rapid and maintained weight loss, that may be amplified in older adults. Despite this, a recent meta-analysis demonstrates that rapid weight loss carries no increased risk of reduction in muscle mass (vs. gradual weight loss) [77].

### Metabolic surgery

The most common procedures performed are Roux-en-Y gastric bypass (RYGB) and sleeve gastrectomy. These procedures are reserved for patients with a BMI ≥ 40 kg/m^2^, or a BMI ≥ 35 kg/m^2^ with obesity-related complications and patients must be refractory to other weight management intervention despite specialist input [78]. Metabolic surgery produces weight loss >30% after 1 year, that can remain >25% after 10 years [79], as well as improving prevalent comorbidities and reducing incidence of T2D, CVD, cancer and mortality [80]. Despite metabolic surgery being among the safest surgical procedures following the introduction of laparoscopic approaches, RYGB and sleeve gastrectomy have greater perioperative complication rates, as well as worse weight reduction and cardiometabolic outcome efficacy, in older (vs. younger) adults, largely explained by underlying CVD [81].

## Evidence for efficacy and safety of approved anti-obesity medicines in older adults

**Fig. 3:**
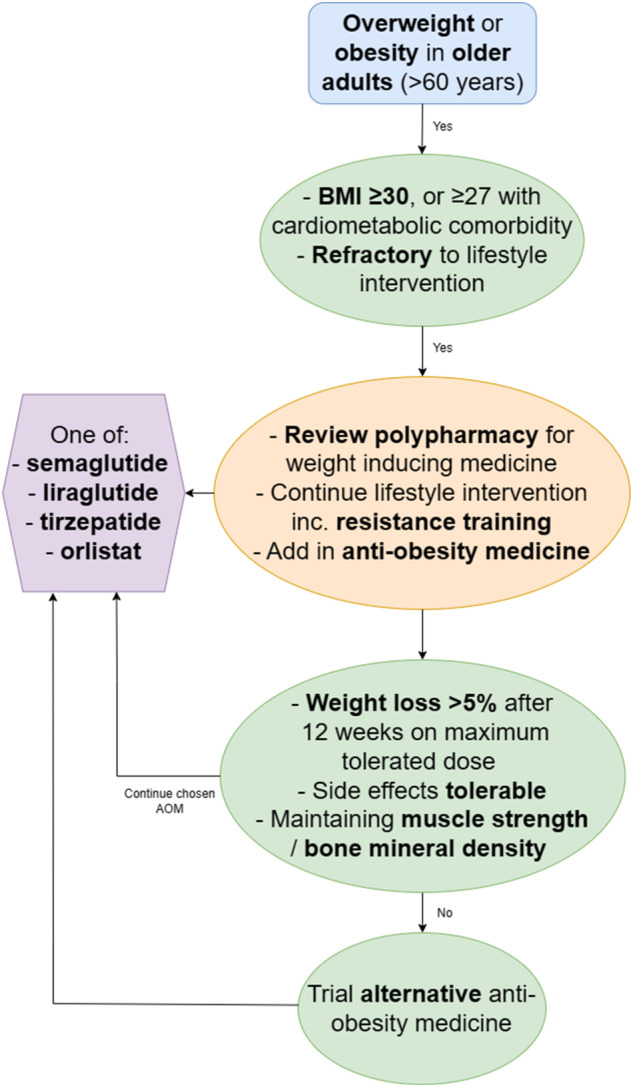
An updated algorithm for the medical management of obesity using approved pharmacological agents. BMI body mass index. If in the USA, phentermine/topiramate could also be considered after semaglutide.

**Fig. 4:**
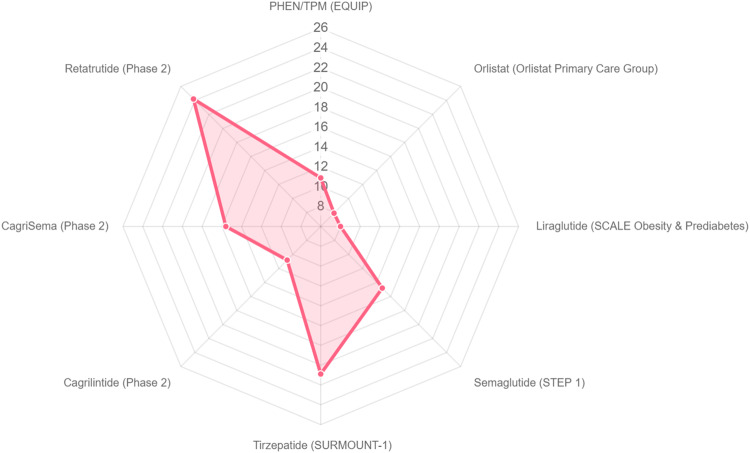
Radar plot demonstrating the percentage weight loss from baseline in the intervention arm of landmark randomised controlled trials of approved and novel anti-obesity medicines that have evidence for use in older adults.

A novel pharmacotherapy algorithm for managing obesity in older adults is proposed in Fig. 3. Data from clinical trials for all approved AOMs with evidence for use in older adults are presented in Table 1, with their mean weight loss presented in Fig. 4. Crucially, many trials may have included older adults, but had an upper age limit in their inclusion/exclusion criteria, resulting in little data for people in their 80s. Moreover, the Summary of Product Characteristics on the Electronic Medicines Compendium states that Therapeutic experience in patients ≥75 years of age is limited. Age cut offs have been included in Table 1. Approved drug dosages are presented in Table 2.

**Table 1:** Table summarising currently approved, and future, anti-obesity medicines with evidence for use in older adults [72, 75, 82, 83, 85, 92–95, 102, 106, 111–113, 115, 116, 119–121].

Drug	RCT (*n*)	BMI (kg/m^2^)	Age (years)	Follow-up (weeks)	Mean weight loss % (≥5% weight loss)	Side effects
Anti-obesity medicines approved in the USA, EU, and UK						
Mixed central and peripheral action						
Liraglutide	SCALE-Obesity and Prediabetes (3731) [83]	38.3	45.1, no upper limit 7% older adults (0.5% >75 years)	56	8 (63.2)	Nausea, diarrhoea, and vomiting, hypoglycaemia, dehydration, deranged renal function, allergic reactions, gallstones, acute pancreatitis
	SCALE-Diabetes (846) [82]	37.2	54.9, no upper limit 7% older adults (0.5% >75 years)	56	6 (54.3)	
	SCALE-Maintenance (422) [72]	35.6	46.3, no upper limit 7% older adults (0.5% >75 years)	56	6.2 (81.4)	
	SCALE-Obesity and Prediabetes extension (2254)	38.3	7% older adults (0.5% >75 years)	160	6.1	
	SCALE-IBT (282) [85]	39	47.2, no upper limit 7% older adults (0.5% >75 years)	56	7.5 (61.5)	
Semaglutide	STEP-1 (1961) [95]	37.9	46, no upper limit	68	14.9 (86.4)	Nausea, dyspepsia, vomiting, flatulence, diarrhoea, abdominal pain, abdominal distention, constipation upper respiratory tract and urinary tract infections, nasopharyngitis, musculoskeletal and connective tissue disorders (back pain), dizziness, headaches, gallstones, acute pancreatitis
	STEP-2 (1210) [94]		46, no upper limit	68	9.6 (68.6)	
	STEP-3 (611) [93]	38	46, no upper limit	68	16 (86.6)	
	STEP-4 Maintenance (803) [92]	38.4	46, no upper limit	20 week run in + 48 weeks	10.6 run in + additional 7.9	
Tirzepatide	SURPASS-2 (1879) [102]	34.2	56.6, no upper limit	40	13 (80)	Nausea, diarrhoea, and constipation
Peripheral action						
Orlistat	Orlistat Primary Care Study Group (635) [106]	36	42.5, no upper limit	52	7.9 (50.5)	Steatorrhea, frequent bowel movements, flatus with discharge, and fecal incontinence (because of non-absorbed fats in the intestine
Anti-obesity medicines approved in the USA						
Central action						
Phentermine/topiramate	EQUIP (1267) [111]	42.2	42.6, upper limit 70 7% older adults	56	9.4 (67)	Dry mouth, paraesthesia, constipation, insomnia, mood and sleep disorders, cognitive impairment, metabolic acidosis, lowers seizure threshold, glaucoma worsening
	CONQUER (2487) [112]	36.6	51.1, upper limit 70 7% older adults	56	8.6 (70)	
	SEQUEL (676) [113]	36.1	52, upper limit 70 7% older adults	108 (52-week extension to CONQUER)	10.5 (79.3)	
Future anti-obesity medicines with evidence in older adults						
Cagrilintide	Phase 2 trial (906) [115]	37.8	52.3, no upper limit	26	10.8 (88.7)	Gastrointestinal disturbance, fatigue
CagriSema	Phase 2 trial (92) [116]		58, no upper limit	32	15.6	Gastrointestinal disturbance, fatigue
Retatrutide	Phase 2 trial (338) [75]		Upper limit 75	48	24.2 (92)	Mild-to-moderate gastrointestinal disturbance
Survodutide	Phase 2 trial (387) [119]		Upper limit 75	46	14.9	Gastrointestinal disturbance
Oral semaglutide	OASIS-1 (667) [120]		No upper limit	68	15.1	As semaglutide
Orforglipron	Phase 2 trial (272) [121]		Upper limit 75	36	14.7	Gastrointestinal disturbance

*EU* European Union.

**Table 2:** Dosing regimen for the approved anti-obesity medicines with evidence for use in older adults.

Drug	Route	Dose
Anti-obesity medicines approved in the USA, EU, and UK		
Mixed central and peripheral action		
Liraglutide	SC	Starting dose of 0.6 mg OD, up-titrated weekly until 3 mg OD
Semaglutide	SC	Starting dose of 0.25 mg, up-titrated to 2.4 mg OW, over 16 weeks
Tirzepatide	SC	Starting dose of 2.5 mg, up-titrated to 15 mg OW
Peripheral action		
Orlistat	PO	120 mg up to 3 times a day, dose to be taken immediately before, during, or up to 1 h after each main meal. 60 mg OD over-the-counter dose is also approved.
Anti-obesity medicines approved in the USA		
Central action		
Phentermine/topiramate	PO	3.75/23 mg for 2 weeks, slowly increased to 7.5/46 mg and a maximum dose of 15/92 mg which is approved after a minimum of 3 months

*EU* European Union, *PO* oral, *SC* subcutaneous, *OD* once daily, *OW* once weekly.

### Mixed centrally and peripherally acting anti-obesity medicines with evidence for use in older adults

Three GLP-1 receptor agonists are currently approved for obesity pharmacotherapy (Tirzepatide is a dual GIP-GLP-1 receptor agonist).

#### Liraglutide

##### Weight loss trials

Satiety and Clinical Adiposity–Liraglutide Evidence (SCALE) trials were comprised of only 7% older adults (0.5% >75 years) [82, 83]. SCALE-Obesity and Prediabetes (overweight/obesity without T2D) randomised 3731 participants to liraglutide 3 mg, or placebo, as an adjunct to lifestyle intervention. Over 56 weeks, participants taking liraglutide 3 mg lost 8% of body weight from baseline (vs. 2.6% with placebo). In total, 63.2% (vs. 27.1% in placebo) had weight loss of ≥5% [83]. Efficacy was reduced in patients with T2D, but still significant compared to placebo [82]. Pooled analysis of participants from SCALE demonstrated similar 5 and 10% weight reduction rates in older and younger adults [84]. In adults of all age who had lost weight with lifestyle intervention initially, liraglutide promoted additional weight loss in SCALE Maintenance [72]. Moreover, liraglutide is able to produce weight loss outside of tertiary health care settings which demonstrates its effectiveness in the real-world [85]. SCALE found improvements in such cardiometabolic risk markers as lipid profile, blood pressure, and glycaemic control [82, 83], consolidated by pooled analysis in older and younger adults [84].

##### Cardiometabolic outcome trials

Prospective study of older adults (mean age 68 years) using liraglutide 3 mg once daily for 24 weeks found improved body composition with reductions in central fat mass, and preserved muscular performance [86, 87], explained by increased myogenesis in myoblasts [88], and the recruitment of skeletal muscle microvasculature [89]. The Liraglutide Effect and Action in Diabetes (LEAD)-3 trial included 746 adults (age up to 79 years) with T2D randomised to liraglutide, 1.2 or 1.8 mg, or glimepiride, and found that weight loss following liraglutide did not adversely impact bone mineral density [90], although we do not have data for the impact of liraglutide at 3 mg.

##### Side effects

Common side effects related to liraglutide use are gastrointestinal disturbance, including nausea, diarrhoea, and vomiting, which may be problematic in older adults. Pooled analysis of SCALE found that older adults had greater gastrointestinal disturbance than younger adults [84]. Despite this, side effects can be mitigated by careful dose titration. Rarer safety concerns that need consideration in older adults are hypoglycaemia, dehydration, acute kidney injury, gallstones, and acute pancreatitis. Importantly for older adults with polypharmacy, absorption of other drugs may be impacted by delayed gastric emptying and gastrointestinal side effects, namely vomiting, associated with GLP-1 receptor agonists [91].

#### Semaglutide

##### Weight loss trials

Semaglutide Treatment Effect in People with obesity (STEP) studies had no upper limit in age [92–95]. STEP-1 took 1961 adults with a BMI ≥ 30 (or ≥27 in people with ≥1 weight-related coexisting condition) without T2D and demonstrated mean weight reduction of 14.8% in participants taking semaglutide 2.4 mg weekly (vs. 2.4% in the placebo group), as an adjunct to lifestyle intervention. 86.4% (vs. 31.5%) of patients achieved weight loss ≥5%, 69.1% (vs. 12%) achieved weight loss ≥10% and 50.5% (vs. 4.9%) achieved weight loss ≥15%. Results were reduced in those with T2D, but still significant (STEP-2) [94]. Further STEP trials have demonstrated mean weight reduction of 16% (vs. 5.7%) when semaglutide was used as an adjunct to intensive behavioural therapy (STEP-3) [93], and improved head-to-head weight reduction compared to liraglutide over 68 weeks, with possible benefits in older adults given the once weekly administration of semaglutide (STEP-8) [96]. STEP-4 gave every participant a 20-week course of semaglutide 2.4 mg once weekly, and produced a mean weight loss of 10.6%, before randomisation to either continued semaglutide treatment or exchange to placebo for an additional 48 weeks. Continuation of semaglutide resulted in a further 7.9% reduction in body weight from their week 20 weight, whereas the placebo group regained 6.9% [92]. However, STEP 1 extension concluded that 1-year withdrawal of semaglutide results in regain of two-thirds of prior weight loss [97], highlighting that withdrawal of semaglutide results in weight regain which may be problematic in older adults who are already using polypharmacy approaches for comorbid disease. Consideration of a cyclical delivery of semaglutide, and other AOMs, could be considered, where semaglutide is administered until a pre-determined body weight is achieved, at which point an interval period is commenced until a pre-determined percentage of weight is regained, before restarting treatment. In addition to being polypharmacologically beneficial, this method would appeal to organisations funding healthcare in the licensing of AOMs. More evidence is needed to clarify the ideal duration that an AOM should be given initially before the first interval, but longer compliance with anti-obesity agents from the outset does result in reduced weight regain following cessation [97]. If this method was to be implemented, it would be imperative to remember the evidence to date suggests benefit only with chronic prescription of respective AOM, both in relation to weight and cardiometabolic disease.

##### Cardiometabolic outcome trials

Pooled analysis of Semaglutide Unabated Sustainability in Treatment of Type 2 Diabetes (SUSTAIN) 1-5 (assessing semaglutide at lower doses of 0.5 and 1 mg doses for the treatment of T2D), found semaglutide had a similar weight reduction, and glycaemic control, efficacy in older and younger adults. In older adult SUSTAIN participants, 37%–59%, and 40%–79%, lost >5% body weight with semaglutide 0.5 mg, and 1.0 mg, respectively (vs. 4%–17% in placebo) [98]. Crucially, Semaglutide Effects on Cardiovascular Outcomes in People with Overweight or Obesity (SELECT) included adults >45 years living with overweight/obesity and established cardiovascular disease (CVD), without T2D, and found Major Adverse Cardiovascular Events (MACE) was reduced by 20% in those taking semaglutide [99]. Lower dose semaglutide (0.1, 0.2 and 0.4 mg) improved non-alcoholic steatohepatitis (NASH) in another trial (mean age 55.9 years, but the inclusion of older adults up to 75 years). There was no improvement in fibrosis and hence results may be explained by weight loss rather than a direct result of semaglutide [100].

##### Side effects

Semaglutide also induces gastrointestinal disturbance, although pooled analysis of SUSTAIN 1-5 found comparable safety profiles in older and younger adults [98]. Rarer side effects include upper respiratory and urinary tract infections, which concerns older adults greater given their enhanced susceptibility to infections [101]. Moreover, musculoskeletal pain and symptoms of the nervous system, such as dizziness and headaches, have been reported, and may exacerbate existing symptoms in older adults. Weight loss which exceeds what is expected in an older adult treated with GLP-1 receptor agonist should be investigated to exclude malignancy.

#### Tirzepatide

##### Weight loss trials

SURMOUNT-1 assessed weight reduction efficacy of increasing tirzepatide doses (vs. placebo) in 2539 participants (mean age 44.9 years, with no upper limit on age). Mean percentage weight change at 72 weeks was 15%, 19.5% and 20.9% with 5, 10 and 15 mg weekly doses of tirzepatide, respectively (vs. 3.1% with placebo). In total, 85%, 89%, and 91% of participants lost 5% bodyweight with 5, 10, and 15 mg of tirzepatide, respectively (vs. 35% with placebo) [102].

##### Cardiometabolic outcome trials

SURPASS-2 demonstrated greater efficacy in the treatment of T2D compared to semaglutide in an older population (mean age 56.6 years, with no upper limit) [103]. Pooled analysis from both SURMOUNT-1 and SURPASS-2 demonstrates reduced MACEs and cardiovascular death, by 48% and 49%, respectively [104].

##### Side effects

Promisingly for older adults, the side effect profile of tirzepatide is reduced, as both glucose-dependent insulinotropic polypeptide (GIP) and GLP-1 receptor agonists can be given at lower doses than when compared to their use in monotherapy.

## Peripherally acting anti-obesity medicines with evidence for use in older adults

### Orlistat

#### Weight loss trials

None of the XENDOS trial participants were older adults, and hence the results from this study may not be applicable to an ageing population, although as the drug works peripherally there is no reason to suggest reduced efficacy or safety in elderly patients. A small pilot study using prospective data from 13 women, between 66–83 years, living with obesity and prescribed orlistat, found weight loss of 9.4% over 6 months [105]. In addition, a 2-year RCT of an older subpopulation in a primary care setting demonstrated similar efficacy of orlistat in both older and younger adults of around 8% [106].

#### Cardiometabolic outcome trials

Older women taking orlistat had improved biventricular diastolic function and beneficial restructuring of their myocardium, producing a lower left ventricular mass, at the end of 6 months, although the cardiac outcomes were likely the result of weight loss rather than independent effect of orlistat [105]. Prospective data from adults >18 years (no upper limit, but mean age only 45 years) found that patients using orlistat had 23% lower rates of myocardial infarction, 32% lower rates of ischaemic stroke and 21% lower rates of new-onset heart failure [107]. These lower outcomes are reflected by improvements in such cardiometabolic risk markers as lipid profile and blood pressure in older adults using orlistat [106].

#### Side effects

Due to decreased dietary fat absorption in patients taking orlistat, drug-drug interactions can occur in older adults taking fat-soluble compounds such as vitamin D, thyroxine, or anticoagulants. Troublesome gastrointestinal disturbance including steatorrhea, frequent bowel movements, flatus with discharge, and fecal incontinence, are the commonest side effects and occur because of non-absorbed fats in the intestine. They can affect ~8% of patients and may result in drug discontinuation [108]. These symptoms may be more troublesome in older adults who suffer from fecal incontinence, and co-prescription of fat-soluble vitamins is needed to ensure adequate nutrition is maintained.

##### Centrally acting anti-obesity medicines with evidence for use in older adults

Only 2% of the Contrave Obesity Research (COR) trials, evaluating naltrexone-bupropion, were older adult participants [109, 110], and hence have not been discussed here.

### Phentermine/topiramate (PHEN/TPM)

#### Weight loss trials

PHEN/TPM combines an amphetamine analogue with a gamma-aminobutyric acid (GABA) agonist, glutamate antagonist, and carbonic anhydrase inhibitor. (1) Controlled-Release PHEN/TPM in Severely Obese Adults (EQUIP) trial (obesity without comorbidities), (2) Controlled-Release Phentermine plus Topiramate Combination in Overweight and Obese Adults (CONQUER) trial (obesity with comorbidities), and (3) SEQUEL (2-year extension of CONQUER), were comprised of 7% of older adult participants across the trials, although the upper age limit was 70 [111–113]. After 56 weeks of treatment in EQUIP, body weight reduction from baseline was 10.9%, and 5.1%, with high (15/92 mg), and low (3.75/23 mg), doses of PHEN/TPM, respectively (vs. 1.6% in the placebo arm). CONQUER demonstrated body weight reduction from baseline of 10.2%, and 7.8%, with high (15/92 mg), and medium (7.50/46.0 mg), doses of PHEN/TPM, respectively, (vs. 1.2% in the placebo arm). SEQUEL demonstrated maintained weight loss over 2 years with 9.3%, and 10.5%, body weight reduction for medium, and high doses, of PHEN/TPM, respectively. A significantly higher proportion of patients achieved ≥5%, 10%, or 15% body weight reduction with PHEN/TPM (vs. placebo) in all trials [111–113]. EQUIP, CONQUER and SEQUEL all demonstrate improvement in such cardiometabolic risk markers as WC, blood pressure, fasting glucose, insulin resistance and lipid profiles [111–113].

#### Side effects

Despite FDA approval, PHEN/TPM is not approved for obesity pharmacotherapy by the EMA due to cardiovascular safety concerns; although one retrospective cohort analysed data from over 500,000 patients taking PHEN/TPM, 36.9% of which were >50 years (no maximum age), and found no increased risk of MACE for current users [114]. PHEN/TPM should be used cautiously in older adults taking hypertension or rate-controlling agents. It is worth noting that EQUIP and CONQUER noted mood and memory disturbance, which may be troublesome in older adults, but commonest side effects were dry mouth, paraesthesia, constipation, and insomnia [111, 112].

## Future anti-obesity agents for use in older adults

Several novel AOMs are currently in development, or undergoing clinical trials, that will likely be available for the treatment of obesity soon, and have included older adult participants (Table 1).

### Cagrilintide

One phase 2 trial demonstrated that weekly injections of cagrilintide induced 10.8% weight loss over 26 weeks, compared to 9% with liraglutide, and 3% with placebo (mean age 52.3 years, with no upper age limit). Gastrointestinal side effects and administration site reactions that were tolerable with slow dose titration, although fatigue appears to be a unique side effect of cagrilintide over GLP-1 receptor agonists [115].

### CagriSema

The combination of cagrilintide and semaglutide. One phase two trial (mean age 58, with no upper age limit) demonstrated mean reduction in body weight of 15.6% in participants taking CagriSema, compared to 5.1% and 8.1% with semaglutide and cagrilintide in monotherapy, respectively, over 32 weeks. Moreover, CagriSema produced greater mean reductions in HbA1c [116]. REDEFINE is an ongoing phase 3 trial acting as market evaluation for both CagriSema and cagrilintide, with 60% randomised to CagriSema, 10% cagrilintide, 10% semaglutide, and 20% placebo, where minimum age at inclusion was 55 years with no upper limit [117].

### Retatrutide

The first triple-incretin agonist (GIP/GLP-1/glucagon) to complete phase 2 development. Two phase 2 trials, including older adults up to 75 years, have recently been published assessing the impact of retatrutide on patients with overweight and obesity, with or without T2D, respectively [75, 118]. In participants with overweight/obesity without diabetes, the highest dose of retatrutide produced 24.2% reduction in body weight after 48 weeks [75]. Efficacy is slightly reduced in those with diabetes, but participants were still able to lose 16.9% of body weight alongside a 2.16% reduction in HbA1c from baseline after 36 weeks [118]. Predicted weight-loss trajectories after the study end points suggests ongoing weight loss with continued therapy. The level of weight loss seen following retatrutide use approaches weight loss noted after metabolic surgery and is the greatest demonstrated from any clinical trial using AOMs to date. Moreover, retatrutide seems to be well tolerated, with the most frequent side effects being mild-to-moderate gastrointestinal disturbance reported in 13–50% of participants [75, 118]. A phase 3 trial is underway.

### Survodutide

A glucagon and GLP-1 receptor dual agonist which has had recent phase two completion, including adults between 18–75 years, BMI ≥ 27 kg/m^2^, without diabetes. Mean weight loss over 46 weeks, at the highest dose of 4.8 mg OD, was 14.9% (vs. 2.8% placebo) [119].

It is also worth acknowledging OASIS 1, which demonstrates mean weight loss of 15.1% (vs. 2.4% placebo) in adult participants (no upper age limit) prescribed oral semaglutide 50 mg OD over 68 weeks [120]. Additionally, the phase 2 trial of orforglipron, a novel non-peptide oral GLP-1 receptor agonist, included adults up to 75 years, and demonstrated mean weight loss of 14.7% (vs. 2.3% placebo) over 36 weeks [121]; with a phase 3 currently underway. Finally, setmelanotide can be used in older adults with monogenic obesity, caused by mutations in the leptin signalling pathway, although this is more commonly initiated in children and younger adults with severe and complex obesity [122].

## Health economics

Older adults living with severe obesity have increased healthcare use and cost [22], although the effect of obesity on years-of-life-lost is greatest in younger adults, and the magnitude of years lost with obesity decreases with ageing [16], irrespective of sex, smoking status, physical activity level, or socioeconomic status [35], complicating the health economic considerations of obesity management in older adults. A National Institute for Health and Care Excellence (NICE) economic model estimated that a £100, 12-week programme, is cost-effective for people living with overweight/obesity if their weight loss is maintained for life, compared with that without intervention; even with weight loss of 1 kg. Similarly, a £200, 24-week programme, is cost effective if weight loss is maintained for life. For programmes costing £500, and £1000, an average additional weight loss of 2, and 3 kg, respectively, must be maintained for life. Crucially, enhanced cost-effectiveness is found in people older than 50, even with weight regain [73]. However, we would suggest that future clinical trials should aim to assess the cost-effectiveness of screening older people with obesity who are undergoing weight management interventions for evidence of sarcopenia, through clinical evaluation including hand grip tests, which are accurate biomarkers for overall strength and function, bone mineral density and consequent fracture risk, and cardiometabolic disease [123].

## Conclusion

Obesity pharmacotherapy in older adults should be encouraged as an adjunct to energy-restriction and physical activity, with an emphasis on resistance training. Anti-obesity medicines, based around incretin receptor agonists and their novel combination with other appetite regulatory targets, have weight reduction efficacy in this population, addressing obesity-related cardiometabolic complications while preserving skeletal muscle mass. The available evidence suggests that older adults derive similar clinical benefits as younger adults with comparable weight loss and an equivalent associated impact on obesity-related complications, with likely improved adherence to treatment regimes. Future randomised controlled trials should specifically address this clinical question in older adults with obesity with an emphasis on efficacy, body composition and health economic modelling.
